# Autofluorescence enhancement for label-free imaging of myelinated fibers in mammalian brains

**DOI:** 10.1038/s41598-021-86092-7

**Published:** 2021-04-13

**Authors:** Irene Costantini, Enrico Baria, Michele Sorelli, Felix Matuschke, Francesco Giardini, Miriam Menzel, Giacomo Mazzamuto, Ludovico Silvestri, Riccardo Cicchi, Katrin Amunts, Markus Axer, Francesco Saverio Pavone

**Affiliations:** 1 European Laboratory for Non-Linear Spectroscopy, University of Florence, Florence, Italy; 2 Department of Biology, University of Florence, Florence, Italy; 3 National Institute of Optics, National Research Council, Rome, Italy; 4 Department of Physics, University of Florence, Florence, Italy; 5 Institute of Neuroscience and Medicine (INM-1), Research Centre Jülich, Jülich, Germany; 6 C. and O. Vogt Institute for Brain Research, University Hospital Düsseldorf, Heinrich-Heine University Düsseldorf, Düsseldorf, Germany

**Keywords:** Biological techniques, Neuroscience, Anatomy, Optics and photonics

## Abstract

Analyzing the structure of neuronal fibers with single axon resolution in large volumes is a challenge in connectomics. Different technologies try to address this goal; however, they are limited either by the ineffective labeling of the fibers or in the achievable resolution. The possibility of discriminating between different adjacent myelinated axons gives the opportunity of providing more information about the fiber composition and architecture within a specific area. Here, we propose MAGIC (Myelin Autofluorescence imaging by Glycerol Induced Contrast enhancement), a tissue preparation method to perform label-free fluorescence imaging of myelinated fibers that is user friendly and easy to handle. We exploit the high axial and radial resolution of two-photon fluorescence microscopy (TPFM) optical sectioning to decipher the mixture of various fiber orientations within the sample of interest. We demonstrate its broad applicability by performing mesoscopic reconstruction at a sub-micron resolution of mouse, rat, monkey, and human brain samples and by quantifying the different fiber organization in control and Reeler mouse's hippocampal sections. Our study provides a novel method for 3D label-free imaging of nerve fibers in fixed samples at high resolution, below micrometer level, that overcomes the limitation related to the myelinated axons exogenous labeling, improving the possibility of analyzing brain connectivity.

## Introduction

The brain shows a complex network of neurons, capable of storing and processing information from a myriad of different inputs regulating cognitive processes and behavior. Individual neurons are interconnected to hundreds or even thousands of other cells forming networks that can extend over large volumes, up to several centimeters for the human brain. The organization of nerve fibers spans many orders of organization: from the microscale of the single axon to the macroscale of the long-range connections connecting far-distant brain regions. Most long-range projecting axons are wrapped by the myelin sheath to permit a reliable and efficient signal transmission^1^.

Reconstructing the intricate organization of these connections remains a challenging goal to achieve due to technical limitations, especially on human brain tissues. Several methods have been developed to map the network of interneuron connections; still, they are limited by the volume that they can analyze (Electron Microscopy)^2^, by the spatial resolution achievable (MRI)^3^ or by the sophisticated equipment needed for the measurement (CARS, THG microscopy)^4^. Optical methods have the potential for scalable large-area high-resolution mapping. For instance, cutting-edge microscopy techniques such as micro-optical sectioning tomography (MOST)^5,6^, serial two-photon tomography (STP)^7^, block-face serial microscopy (FAST)^8^, and light sheet microscopy (LSM)^9,10^, permit the reconstruction of large volumes of tissue and even entire organs with micrometer resolution. However, they need a source of contrast to detect the structure of interest. In this respect, myelin staining is an unmet technical challenge. Exogenous dyes^11^ are used to stain fibers composing the white matter, but nonspecific binding and inefficient diffusion of dyes hinder single fiber imaging in large volumes. Transgenic models^12^, injection of virus^13,14^ or injection of dyes^15^ can be used for specific labelling of circuits of interest. Nevertheless, with these approaches the analysis is confined to animal models where gene manipulation can be performed and virus/dyes can be administrated in vivo. Moreover, the necessity of prelabeled conditions prevents the possibility of studying archived samples.

To overcome these limitations, we developed MAGIC (Myelin Autofluorescence imaging by Glycerol Induced Contrast enhancement), a simple label-free method that opens the possibility of performing sub-micron resolution fluorescence imaging of myelinated fibers in 3D at the mesoscale level of different mammalian brains. MAGIC is a methodology that permits, with a glycerol-based procedure, to enhance the autofluorescence of myelin of fixed samples. We characterized and validated the protocol using different methods, including Fluorescence-Lifetime Imaging Microscopy (FLIM) and Raman spectroscopy. MAGIC is compatible with conventional immunostaining and can be used in combination with conventional fluorescence microscopy techniques, such as confocal and two-photon fluorescence microscopy. We demonstrated the widespread applicability of the methodology by investigating neuronal filament organization in 3D in different mammalian brains such as mouse, rat, vervet monkey, and humans.

Finally, to quantitatively analyze the 3D reconstructions of the tissue structure, we implemented a tool based on Structure Tensor Analysis, and we successfully investigated the orientation abnormalities of the fibers in control and Reeler mouse hippocampi treated with MAGIC. The Reeler mouse (Reelin deficient—RELN-/- Reeler) is a well-known animal model for several neurological and neurodegenerative disorders^16^ and presents severe disruption of cells and fibers organization^17^. Our approach permits to investigate the tissue organization in 3D, allowing for a specific evaluation of the alteration in the different areas of the hippocampi.

In the present work, we overcame the restrictions imposed by current techniques for myelinated fiber analysis, developing a protocol to successfully investigate neuronal projection at sub-micron resolution through 3D reconstructions of large volumes with fluorescence optical microscopy.

## Results

### The MAGIC protocol implementation

**Figure 1:**
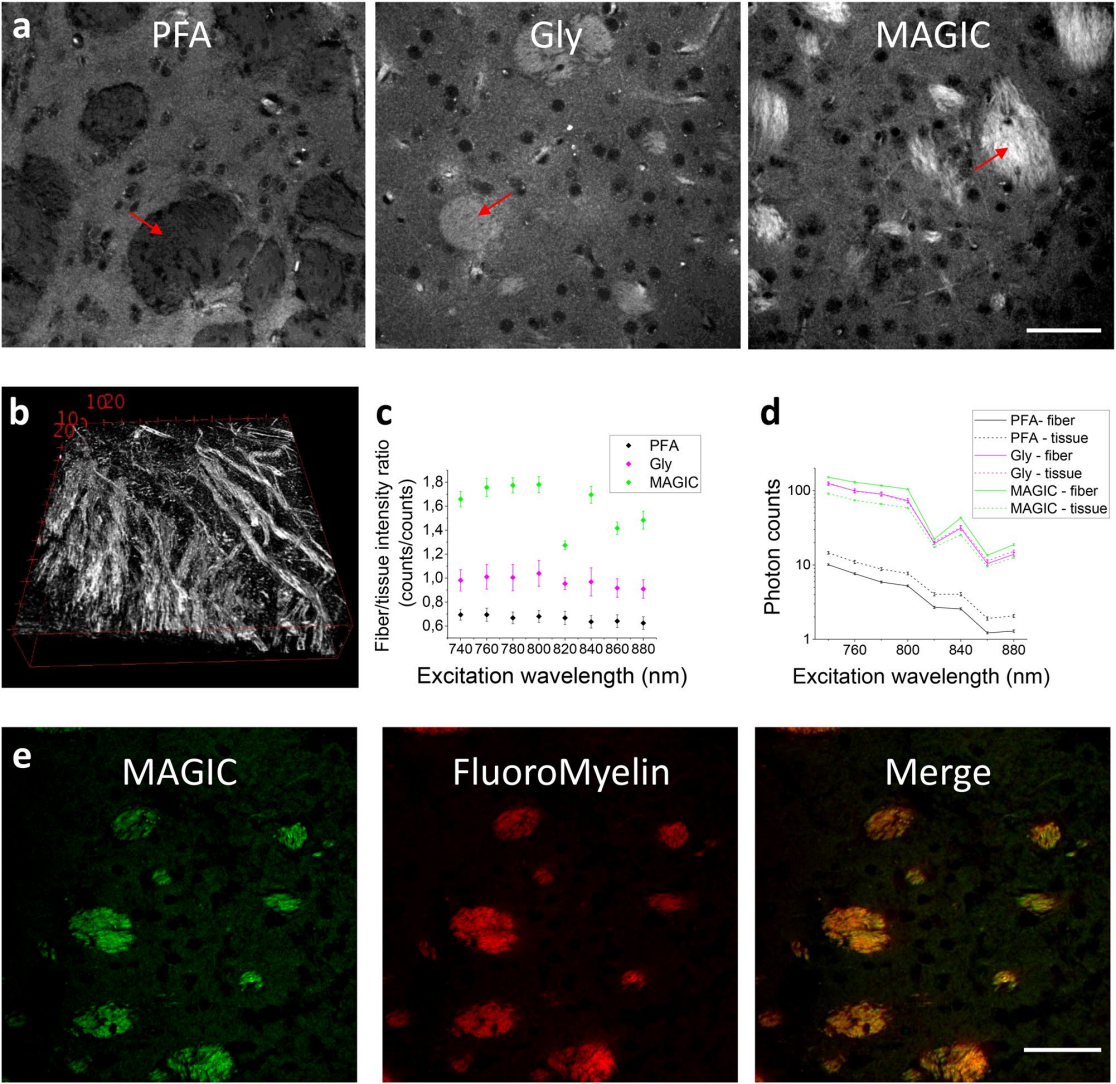
The MAGIC protocol. (**a**) Representative TPFM images of a mouse brain section during the three subsequent steps of the MAGIC protocol: fixation (PFA), glycerolization (Gly), and washing (MAGIC). The images show different areas of the caudate putamen. For each condition, a fiber bundle is indicated with a red arrow. Scale bar = 50 μm. (**b**) 3D reconstruction of myelinated fibers, imaging performed with TPFM at the resolution of (0.44 × 0.44 × 1) μm^3^. Box scale = 10 μm. (**c**) Measurement (mean ± std.err) of photons emitted by the myelinated fibers (fiber) and the surrounding tissue (tissue) during the different steps of the protocol (PFA, Gly, MAGIC) detected at different excitation wavelengths. (**d**) Intensity contrast (mean ± std.err) observed at different excitation wavelengths during the three steps of the MAGIC protocol. (e) Images of a mouse brain section treated with MAGIC and labeled with FluoroMyelin red. In green and red respectively, the autofluorescence and the exogenous signals are shown. Images were obtained with TPFM, scale bar = 50 μm. Images and the 3D rendering were prepared using Fiji (www.fiji.sc/Fiji) ^20^, graphs were prepared using OriginPro 9.0 (www.originlab.com).

Glycerol has been widely used as a mounting and refractive index matching medium because of its biocompatibility^18^. We implemented the MAGIC protocol from the observation that glycerol removal from previously fixed and embedded tissue allows for a specific enhancement of myelin autofluorescence. MAGIC includes three steps (Fig. 1a): fixation with paraformaldehyde (PFA), embedding in glycerol (Gly), and removal of glycerol by washing in saline solution (MAGIC). During the procedure, myelinated fibers undergo a specific increase of fluorescence signal, allowing for high-resolution 3D reconstruction of the axons (Fig. 1b, Supplementary video SV1). The protocol did not introduce any exogenous fluorophores and is based on the local enhancement of autofluorescence from endogenous molecules. Indeed, the number of emitted photons rises significantly during the different steps of the protocol (Fig. 1c). Moreover, in PFA, we observed a negative fluorescence contrast between fibers and surrounding tissue; instead, after the MAGIC protocol, the contrast becomes positive and is significantly enhanced due to the increase of myelinated fiber autofluorescence signal (Fig. 1d). To demonstrate that the fluorescence signal is emitted by myelinated fibers, we labeled on a mouse brain section treated with MAGIC with a myelin-specific exogenous dye: the FluoroMyelin red^19^. The signal emitted by the dye perfectly overlaps the autofluorescence signal of the fibers, indicating that the fluorescence is indeed coming from myelin (Fig. 1e).

### Fluorescence and Raman characterization of the protocol

**Figure 2:**
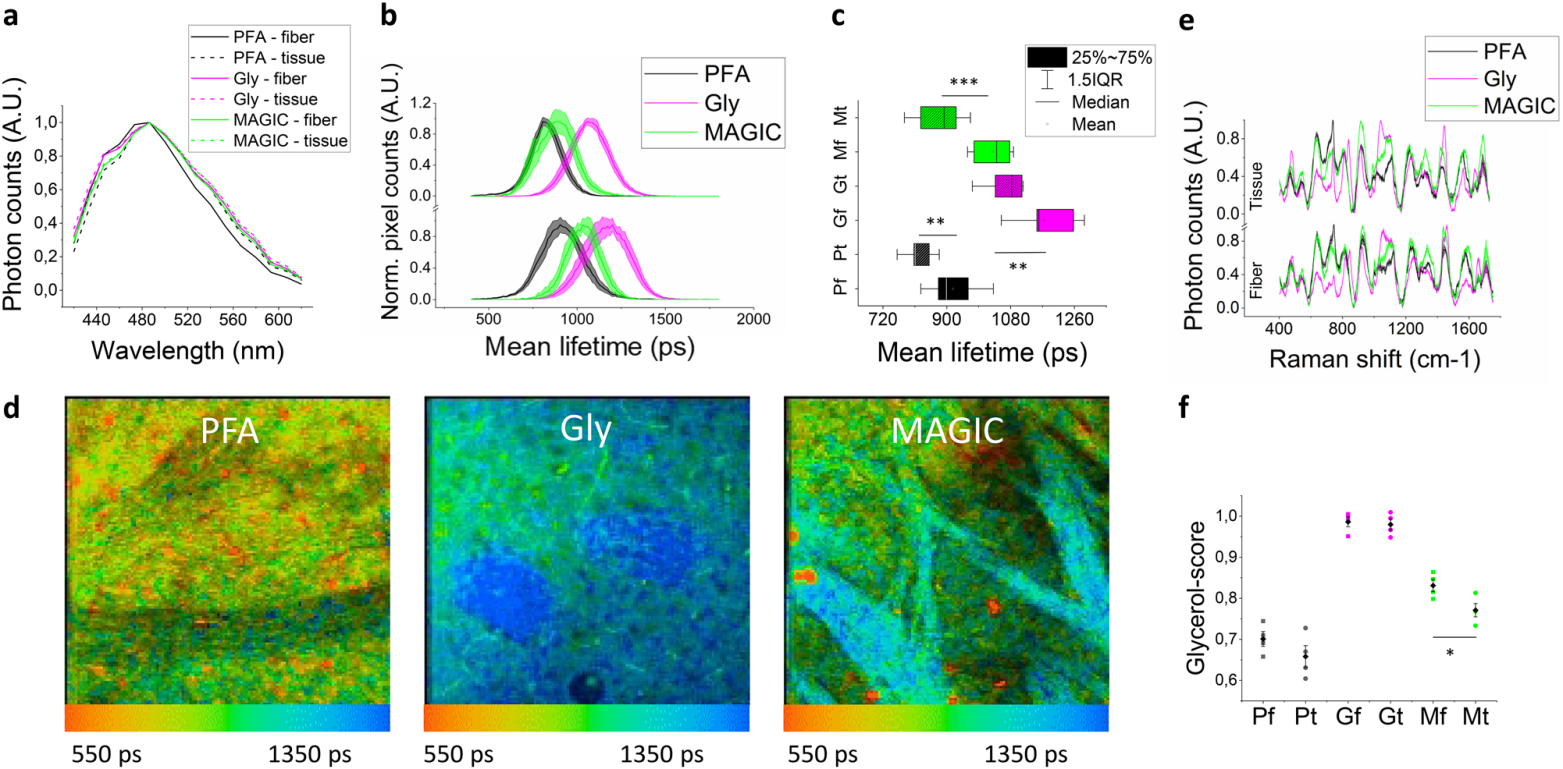
Characterization of the protocol. (**a**) Fluorescence emission spectra of the different samples excited with TPFM at 800 nm. (**b**) Fluorescence lifetime distributions (mean ± std.err) of fiber (bottom) and tissue (top) during the MAGIC steps. (**c**) Box chart plot of fluorescence lifetime values. (**d**) FLIM representative images during the three steps of the MAGIC protocol. Lifetime color scale is set from 550 to 1350 ps. (**e**) Raman spectra (mean ± std.err) of fiber and tissue during the MAGIC steps. (**f**) Glycerol scores (mean ± std.err) calculated along with the three major bands (550, 850, and 1465 cm^-1^) of the glycerol Raman spectrum. Statistical significance: *< 0.05 **< 0.001 *** < 0.0005. Images were prepared using SPC Image 4.9.7 (www.becker-hickl.com), graphs were prepared using OriginPro 9.0 (www.originlab.com).

The enhancement of myelin autofluorescence resulting from the protocol has been studied through the combined analysis of both spectral- and time-resolved TPF and Raman microscopies, as these techniques can provide information about fluorophore concentration, molecular environment, and composition. The spectral analysis of fluorescence signals collected from mouse brain sections revealed that the emission spectrum is not altered throughout the steps of the protocol (Fig. 2a).

To further investigate the origin of the phenomenon, we performed a time-resolved analysis of the emitted photons with FLIM^21^. An increase in fluorescence lifetime can be observed after glycerolization on both fibers and surrounding tissue, although MAGIC modifies differently the dynamics of their fluorescence decays. Following deglycerolization, the fluorescence lifetime of brain tissue decreases at values comparable to those ones obtained from PFA samples (with only 60 ± 30 ps average difference), while that of myelin fibers remains at significantly higher values, resulting in a 110 ± 40 ps difference from the corresponding PFA mean lifetime (Fig. 2b-d). These findings suggest that different changes in the molecular environment surrounding the fluorescent molecules occur inside and outside the fibers. Additional characterization of the protocol was obtained using Raman spectroscopy^22^ as a tool to probe molecular content and prove the involvement of glycerol in the fluorescence enhancement of myelinated fibers (Fig. 2e). In fact, glycerolized tissue spectra are characterized by the presence of glycerol Raman peaks around 485, 550, 850, 925, 1060, and 1465 cm-1 (Supplementary figure S1), as compared to PFA samples; such spectral signatures typical of glycerol disappear in the surrounding tissue after MAGIC, whereas they are preserved within myelinated fibers. Conversely, no DMSO contributions (present in the first step of the protocol to enhance glycerol penetration) were detected from the Raman spectra (Supplementary figure S1), indicating that glycerol is the only substance that significantly affects the molecular composition of the examined tissues. A more detailed analysis based on the main Raman bands of glycerol (as described in the Methods section) was performed for quantitatively evaluating the involvement of glycerol in the process (Fig. 2f). After MAGIC, myelinated fibers show, on average, a significantly smaller decrease (~ 16%) in the intensity of glycerol-related Raman peaks with respect to the surrounding tissue (~ 21%). These results indicate that glycerol plays a central role in the MAGIC protocol. The higher fluorescence lifetime and fluorescence intensity measured in the fibers suggests an anti-quenching effect, and Raman spectroscopy proves the involvement of glycerol in the fluorescence emission of myelinated fibers. Therefore, a possible explanation for this phenomenon is that the fluorescence enhancement could be due to the glycerol that remains confined inside the myelinated fibers after MAGIC due to its higher affinity^23,24^ to this structure with respect to the surrounding tissue.

### Application on rodent and primate brains

MAGIC is highly versatile and can be successfully applied to a wide variety of samples. The fluorescence emitted from myelinated fibers can be detected not only with two-photon excitation but also with conventional one-photon microscopy. Supplementary figure S2 displays images acquired with a commercial confocal microscope at various excitation wavelengths (405, 488, and 561 nm) and from four different species: mouse, rat, vervet monkey, and human (Supplementary figure S3). Using a high magnification objective (Nikon 100 × immersion oil objective), it was possible to study the different organizations of the myelin sheaths which surround the axons. The high contrast obtained with the MAGIC methodology allowed measuring both the inner and the outer diameters of the myelinated axons, as shown in Supplementary figure S4. In addition to label-free detection of fibers, we found that the MAGIC protocol is compatible with conventional immunofluorescence, allowing us to perform multicolor imaging of different structures. In particular, after staining human brain sections treated with MAGIC with an anti-GFAP antibody, we could successfully image astrocytes in both gray and white matter, as shown in Supplementary figure S5, and in Supplementary videos SV2 and SV3.

**Figure 3:**
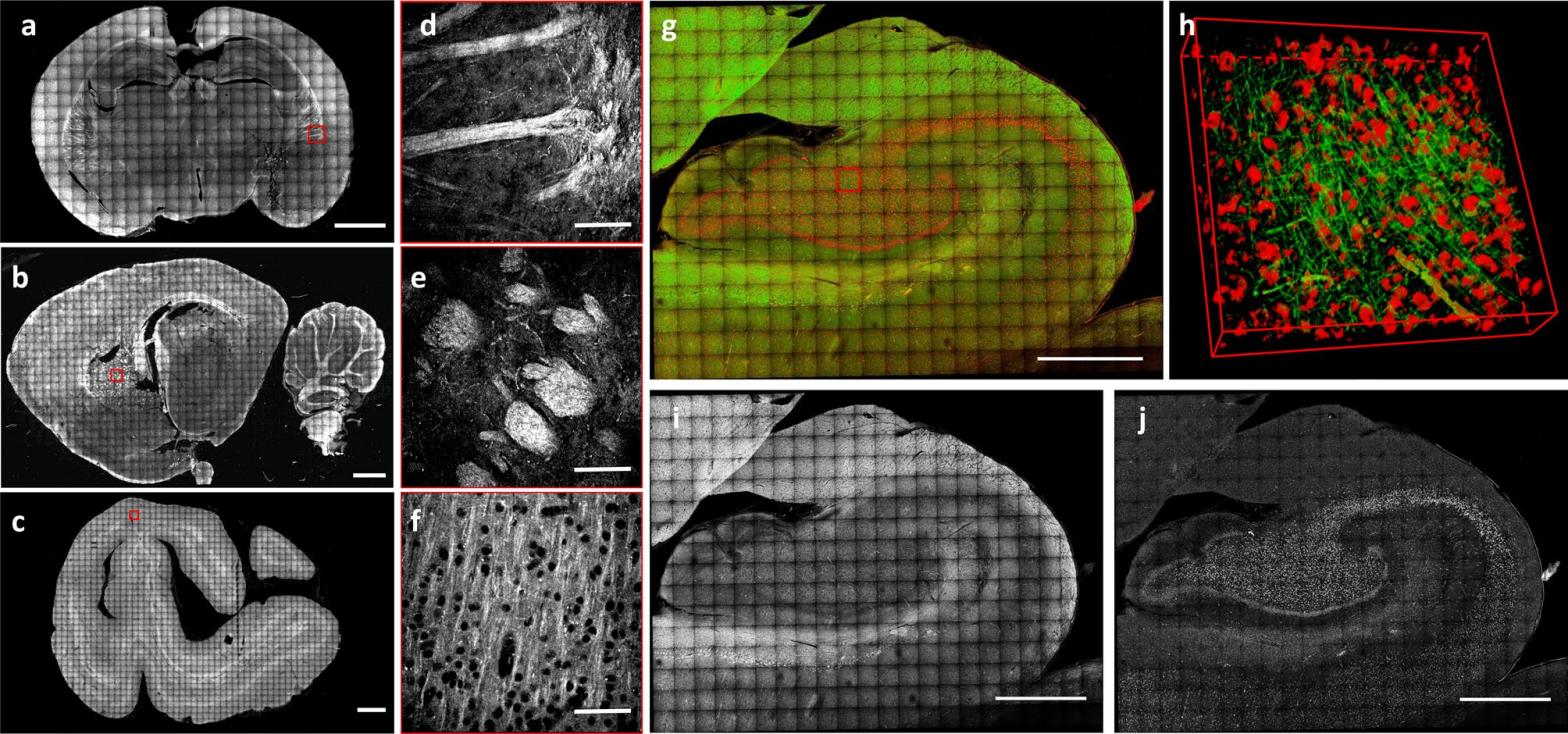
High-resolution reconstruction. (**a**–**c**) Maximum intensity projection (MIP) of the reconstruction of 60-µm-thick brain sections treated with MAGIC: mouse (**a**), rat (**b**), and vervet monkey (**c**), respectively. Scale bar = 1 mm. (**d**–**f**) Magnified inset corresponding respectively to the red boxes in **a**, **b**, **c**. Scale bar = 50 μm. (**g**) MIP of the mesoscale reconstruction of a human hippocampus 60-µm-thick coronal section. Scale bar = 1 mm. (**h**) 3D rendering (450 × 450 × 60 µm^3^) of the stack indicated by the red box in g. (**i**) Green channel showing the myelinated fibers enhanced by MAGIC. (**j**) The red channel of the MIP in **g** showing the autofluorescence of the cell bodies produced by lipofuscin pigments. Images and the 3D rendering were prepared using Fiji (www.fiji.sc/Fiji)^20^.

Finally, in order to demonstrate the possibility of using MAGIC for connectomic analysis, mesoscopic reconstruction of 60-μm-thick brain sections from different mammal species were obtained using TPFM. A coronal mouse slice of about 1 × 0,7 mm^2^ (Fig. 3a, d), a sagittal rat slice of about 2 × 1,1 mm^2^ (Fig. 3b, e), a coronal vervet monkey slice of about 2 × 1,5 mm^2^ (Fig. 3c, f), and, more importantly, a coronal human slice of the hippocampal region (about 1 × 0,7 mm^2^; Fig. 3g–j) were imaged after being treated with the MAGIC protocol. The reconstructions permitted to visualize various anatomical structures of the brains with submicron resolution. Interestingly, in the human brain sample, the presence of lipofuscin pigments^25^ allowed, in combination with MAGIC, both label-free detection and 3D reconstruction of both neuronal fibers and cell bodies (Fig. 3i, j, and Supplementary video SV4), obtaining a more comprehensive information on the anatomical organization of the human hippocampus.

### Fibers' organization in control and Reeler mouse hippocampi

**Figure 4:**
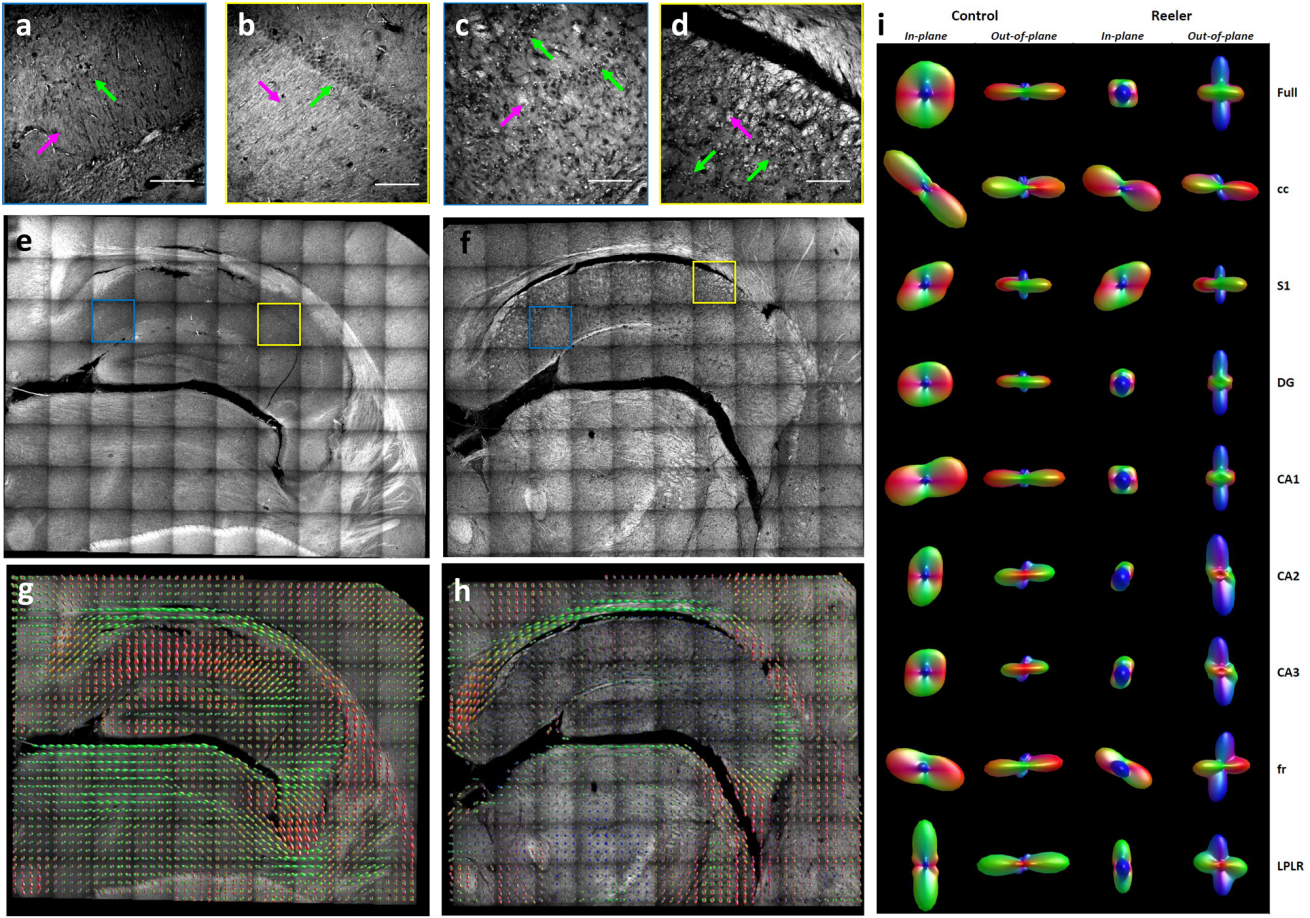
Fibers' orientation in Control and Reeler Hippocampi. (**a**-**d**) Magnified insets of blue and yellow boxes in **e** and **f**, respectively. Green arrows point to neuronal cell bodies, magenta arrows to myelinated fibers. Scale bar = 50 μm. (**e**, **f**) MIP of the mesoscale reconstructions of a control and Reeler mouse hippocampus 60-µm-thick coronal section. Scale bar = 1 mm. (**g**, **h**) Images show the ODF maps obtained from analyzing the full 3D hippocampus reconstruction of the control and the Reeler mouse, sampling 16 vectors for each ODF. (**i**) In-plane and out-of-plane orientation of the single ODF obtained by analyzing all the vectors of the full mosaic reconstructions and each of its ROIs. Acronyms list = cc: Corpus Callosum; S1: Primary Somatosensory Cortex; DG: Dentate Gyrus; CA1, CA2, CA3: field CA1, CA2, CA3 of hippocampus; fr: Fasciculus Retroflexus; LPLR: Lateral Posterior Thalamic Nucleus. Images were prepared using Fiji (www.fiji.sc/Fiji) ^20^while ODF representations using MRtrix3 (https://mrtrix.readthedocs.io/en/latest/)^26^.

In order to demonstrate that the MAGIC protocol allows characterizing the 3D anatomical tissue organization, we compared the structural organization of different regions in the hippocampus of both a control and a Reeler mouse. We applied the method to a 60-μm-thick brain section (stereotactic coordinate: -2.18 mm Bregma; 1.62 mm Interaural) from a control (Control) and a Reeler mouse (Reeler). Imaging of the right hippocampal region was performed with TPFM at a resolution of 0.44 × 0.44 × 1 μm^3^. We found that in the Reeler sample, the organization of neuronal cell bodies and fibers was different compared to that of the control mouse. The Reeler mouse showed a duplication and disorganization of the pyramidal cell layer, moreover, it displayed disruption of the organization of the fibers that intermingled and grouped in abnormal bundles (Fig. 4a-d).

To quantify the observed alteration on the mesoscale reconstruction (Fig. 4e, f), different regions of the hippocampus (cc: Corpus Callosum; S1: Primary Somatosensory Cortex; DG: Dentate Gyrus; CA1, CA2, CA3: field CA1, CA2, CA3 of hippocampus; fr: Fasciculus Retroflexus; LPLR: Lateral Posterior Thalamic Nucleus) were selected and segmented according to their anatomical classification (Supplementary figure S6). Then, to compare the anatomical orientation of the two different specimens, we implemented a custom-made automatic Structure Tensor Analysis^27^ tool followed by an Orientation Distribution Function (ODF)^28^ evaluation of the derived vectors that we applied to the full reconstruction and to each region of interest (ROI), (Fig. 4g-i, Supplementary figure S7). We found that the primary glyph orientation of the control mouse is mostly constituted by in-plane contributions in all the selected ROIs, while six out of eight areas of the Reeler sample were distributed in the out-of-plane direction (Fig. 4i and Table 1). These findings demonstrate that the inner hippocampal connectivity of the Reeler mouse differs significantly from the fiber organization of a normal mouse.

**Table 1:**
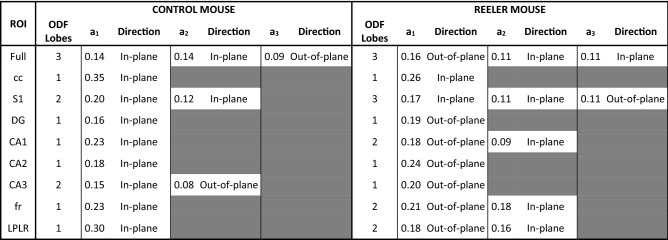
ODF components analysis

Amplitude (a1, a2, a3) and main orientation (direction) of the significant ODF components of both control and Reeler mouse hippocampi are shown. Table cell is gray if the peak amplitude is < 50% of the primary peak. Acronyms list = Full: full field of view; S1: Primary Somatosensory Cortex; cc: Corpus Callosum; CA1, CA2, CA3: field CA1, CA2, CA3 of hippocampus; DG: Dentate Gyrus; fr: Fasciculus Retroflexus; LPLR: Lateral Posterior Thalamic Nucleus.

## Discussion

The present study introduces MAGIC (Myelin Autofluorescence imaging by Glycerol Induced Contrast enhancement), a three-steps glycerol-based procedure that enhances the autofluorescence of myelin, enabling to perform homogeneous label-free fluorescence staining of neuronal fibers in different mammalian brains. We characterized the protocol using different methods, including FLIM and Raman spectroscopy, demonstrating that the fluorescence signal is enhanced by the glycerol residuals that are retained by the myelin sheaths after the washing step, and not by the surrounding tissue. Indeed, previous work studying glycerol interactions with biological macromolecules explains its affinity with membranes as results of different conditions^24^: the so-called preferential hydration^29^ (or preferential exclusion of glycerol), preferential interactions at the membrane–solvent interface^30^, and the partitioning of glycerol into the membrane^31^. In the preferential hydration condition proteins show a higher affinity of the surface for water than that for glycerol. This behavior can be associated with the observation that the glycerol adjacent to the proteins in the cytosol is washed away during the third step of MAGIC. On the other hand, the preferential interactions at the membrane–solvent interface, together with the partitioning of glycerol into the membrane, explain the observed retainment of the glycerol in the myelin sheath, a membrane rich of membrane bilayers.

A significant advantage of the MAGIC protocol is its widespread applicability due to the fact that glycerol is a very common biocompatible mounting medium for tissue samples. MAGIC has a broad spectrum of application as shown by imaging and large-area 3D mesoscopic reconstruction of mouse, rat, vervet monkey, and human brain samples, with both confocal and TPFM at various wavelengths. MAGIC is performed ex-vivo and does not require pre-mortem treatment, enabling the study of fixed archived tissues. Our protocol could provide details of myelin substructures visible under high-magnification objective. By performing staining with an anti-GFAP antibody, we also demonstrated the compatibility with conventional immunofluorescence.

Finally, to enable quantitative analysis on the organization of fibers on the 3D reconstruction obtained with MAGIC in combination with TPFM imaging, we developed a custom-made automatic Structure Tensor Analysis tool followed by an Orientation Distribution Function evaluation of the derived vectors, and we applied it to analyze the tissue structure of a control and Reeler mouse hippocampi.

The methodology presented here demonstrates the possibility of reconstructing the organization of myelinated fibers over large volumes in 3D at sub-micron resolution, enabling the study of the brain anatomy in both physiological and pathological conditions offering a reliable method for integrated quantitative analyses. Nevertheless, MAGIC suffers some limitations. For instance, it cannot be used for in-vivo studies or on intact organs since it requires post-mortem fixation and slicing. In the future, a combination with a faster optical technique, such as light-sheet microscopy, could facilitate scaling up the connectome analysis. Moreover, the association with specific neuronal type labeling could provide single axon tracing. Because MAGIC is non-destructive (i.e., it results in a series of preserved brain slices) it could be combined with other techniques such as 3D-PLI^32^, histological evaluation or gene expression analysis by in situ hybridization techniques.

To conclude, the versatility and simplicity of MAGIC will allow easy implementation of the technique in many laboratories, offering the possibility of using 3D investigation for routine examinations. We thus believe that MAGIC will help to have a more profound comprehension of the brain structure, bringing a significant impact on neuroscience.

## Methods

### Specimen collection

The investigated brain sections were obtained from postmortem brains from different species: mouse (control C57BL/6 and Reelin-deficient mouse model—RELN-/- Reeler, male, six months old), rat (Wistar, male, three months old), vervet monkey (African green monkey: Chlorocebus aethiops sabaeus, male, between one and two years old), and human (male, 87 years). The procedures for rodents were approved by the institutional animal welfare committee at Forschungszentrum Jülich GmbH, Germany, and were in accordance with the European Union guidelines for the use and care of laboratory animals. The vervet monkey tissue sample was acquired in the project "Postnatal development of cortical receptors and white matter tracts" (4R01MH092311-05) funded by the NIMH of the National Institutes of Health. The project was carried out in accordance with the UCLA Chancellor's Animal Research Committee ARC #2011–135 and by the Wake Forest Institutional Animal Care and Use Committee IACUC #A11-219 that approved the experimental protocols. The human brain was acquired in accordance with the ethics committee at the Medical Faculty of the University of Rostock, Germany #A2016-0083. Body donors gave written informed consent for the general use of postmortem tissue used in this study for aims of research and education. The usage is covered by a vote of the ethics committee of the medical faculty of the Heinrich Heine University Düsseldorf (#4863). All methods were carried out in accordance with relevant guidelines and regulations.

### MAGIC preparation protocol

Brains from different species (mouse, rat, vervet, and human) were fixed with 4% paraformaldehyde (PFA) solution at 4 °C for several weeks (human brain: > 3 months, vervet, rat, and mouse brains: 1–2 weeks). The brains were embedded first in a 10% glycerol, 2% DMSO, 4% formaldehyde solution at + 4 °C, then in a 20% glycerol, 2% DMSO, 4% formaldehyde solution at + 4 °C for mouse and rat brains 7 days in total, while for vervet and human brains ≥ 3 weeks. After treatment with 2% dimethyl sulfoxide for cryoprotection, brains were dipped in cooled isopentane (-50 °C) for several minutes (mouse and rat brains: > 5 min, human and vervet brains: > 30 min). The frozen brains were cut with a cryostat microtome (Leica Microsystems, Germany) at a temperature of -30 °C into sections of approximately 60 μm thickness. Brains were cut along one of the three mutually orthogonal anatomical planes: coronal, horizontal, or sagittal. Finally, brain sections were incubated in a Phosphate Buffer Saline solution (PBS) 0.01 M at room temperature (RT) for a month for mouse and rat brains and three months for vervet and human brains. Before imaging, the sections were mounted with PBS and coverslipped. For myelin characterization, the labeling was performed using the FluoroMyelin red dye (Thermo Fisher Scientific, cat. num. F34652): mouse brain sections were incubated in a solution of 1:300 FluoroMyelin in PBS for 20 min at room temperature. Then sections were rinsed in PBS 3 times for 10 min each.

### Fluorescence microscopy imaging

Fluorescence images were obtained using a commercial confocal microscope (Nikon Eclipse TE300 C2 LSCM, Nikon, Japan) equipped with a Nikon 60 × or 100 × immersion oil objective (Apo Plan, NA 1.4), and a custom-made two-photon fluorescence microscope (TPFM). Briefly, a mode-locked Ti: Sapphire laser (Chameleon, 120 fs pulse width, 90 MHz repetition rate, Coherent, CA) operating at 800 nm was coupled into a custom-made scanning system based on a pair of galvanometric mirrors (LSKGG4/M, Thorlabs, USA). The laser was focused onto the specimen by a refractive index tunable 25 × objective lens (LD LCI Plan-Apochromat 25X/0.8 Imm Corr DIC M27, Zeiss, Germany). The field of view was 450 × 450 μm^2^, the resolution employed was 0.44 × 0.44 μm^2^ or 1.75 × 1.75 μm^2^ for high- and low-resolution reconstruction, respectively. The system was equipped with a closed-loop XY stage (U-780 PILine XY Stage System, Physik Instrumente, Germany) for the radial displacement of the sample and with a closed-loop piezoelectric stage (ND72Z2LAQ PIFOC objective scanning system, 2 mm travel range, Physik Instrumente, Germany) for the axial displacement of the objective. The fluorescence signal was collected by an independent GaAsP photomultiplier module (H7422, Hamamatsu Photonics, NJ). Emission filters of 482/35 nm and 618/50 nm were used for fibers and cell body detection, respectively.

### Mesoscale reconstruction

To perform mesoscale reconstruction of the samples, the volume of interest was acquired with TPFM performing z-stack imaging of adjacent regions. Each stack had the depth equal to the thickness of the Sect. (60 μm) with a z step of 1 or 2 μm between images. The overlap of adjacent stacks was set to 40 μm. The stitching of all the acquired stacks was performed using ZetaStitcher (G. Mazzamuto, "ZetaStitcher: a software tool for high-resolution volumetric stitching" https://github.com/lens-biophotonics/ZetaStitcher). Low-resolution reconstructions were performed on the mouse coronal section, the rat sagittal section, and on the vervet section. High-resolution imaging was performed on the human hippocampus section and on the Reeler and control coronal sections.

### Photons counting and Fluorescence Lifetime Imaging Microscopy (FLIM) measurements

FLIM measurements were performed on mouse brain sections (N = 15) in different conditions (PFA = 4, Gly = 6, and MAGIC = 5) using a custom-made multimodal setup^33^. The collected fluorescence was sent to a high-speed PMT for photon counting PMH-100 (Becker-Hickl GmbH, Berlin, Germany) and then processed by a single-photon counting FLIM board SPC-730 (Becker-Hickl GmbH) for time-resolved analysis. Analysis of the obtained FLIM images was performed using the software SPC Image 4.9.7 (www.becker-hickl.com, Becker-Hickl GmbH, Berlin, Germany), fitting the fluorescence decay data with a double-exponential decay function. From each image, we selected ROIs (10 for PFA, 12 for Gly, 8 for MAGIC) corresponding to the myelin sheath and the surrounding brain tissue in order to analyze the distribution of their lifetime values separately. The fluorescence intensity from the same samples was also measured using a photon-counting approach in order to evaluate the fluorescence efficiency of the MAGIC protocol. We measured the fluorescence intensity at different excitation wavelengths (from 740 to 880 nm), and we normalized it to the square of the corresponding laser power. For each condition, we selected the maximum amount of 240 μm^2^ ROIs detectable from the sample: 43 and 56 for PFA tissue and fibers respectively, 49 and 38 for Gly, 68, and 62 for MAGIC. Contrast evaluation was performed by dividing the fluorescence intensity detected from myelin fibers by that of the surrounding tissue.

### Fluorescence spectral measurements

The multimodal microscope, using an excitation wavelength of 800 nm, was used to perform the spectral analysis measurements. Autofluorescence signals were collected from samples (N = 3) during the different conditions of the protocol: fixation (PFA), glycerolization (Gly), and washing (MAGIC). The collected fluorescence signal was coupled to a multimode optical fiber by means of a 10 × objective lens (Nikon, Tokyo, Japan) and detected in the 420–620 nm range using a multispectral detector PML-Spec (Becker-Hickl GmbH, Berlin, Germany) with 16 spectral channels. Each channel recorded a fluorescence intensity image, from which we selected regions of interest (ROIs) corresponding to the myelin sheath (N = 3) and the surrounding brain tissue (N = 3) in order to analyze the intensity of their fluorescence emission separately.

### Raman measurements

We used a commercial Raman microscope (XploRA INV, Horiba, Kyoto, Japan) with λ_EX_ = 532 nm for collecting the Raman spectra of mouse brain tissues fixed in PFA, glycerolized, and after the MAGIC protocol (N = 4 for each condition). For each acquisition, we used a 60 × objective (Nikon, Tokyo, Japan) for scanning a 10-µm-area while recording the Raman signal between 400 and 1750 cm^-1^ on a cooled CCD camera coupled to a spectrograph with a 1800 lines/mm grating: each measurement lasted 30 s. The recorded spectra were processed for removing the fluorescence signal through an automated iterative routine (Vancouver Raman Algorithm). Each resulting Raman spectrum was normalized to its maximum intensity. Then, in order to evaluate the glycerol content within the examined tissue areas, we performed a spectral projection of all Raman spectra along with three major bands (550, 850 and 1465 cm^-1^) of glycerol. In particular, we calculated the scalar product between the acquired spectra and the Raman peaks recorded from the glycerolized mounting medium solution. This allowed obtaining a score for each acquired spectrum ("glycerol score"), which is equal to 1 for glycerolized tissues and < 1 otherwise.

### Data analysis

Graphs and statistical analyses were done with OriginPro 9.0 (OriginLab Corporation: www.originlab.com). Mean and standard errors are displayed for each chart. Statistical analyses were performed using a two-sample t-test. Stacks and 3D stitched volume renderings were obtained using Fiji (www.fiji.sc/Fiji)^20^, videos were performed using Amira 5.3 (ThermoFisher Scientific: www.thermofisher.com/it/en/home/industrial/electron-microscopy/electron-microscopy-instruments-workflow-solutions/3d-visualization-analysis-software/amira-life-sciences-biomedical.html)^34^.

### STA evaluation

Following the preprocessing operations detailed in the Supplementary information, a Structure Tensor Analysis (STA) was conducted at 5 µm spatial resolution on the whole mesoscale reconstruction of the hippocampus and separately on the selected ROIs (Fig. 4 and Supplementary Fig. 6) for estimating the local brain tissue orientation. To this end, we employed a custom STA tool developed by our laboratory in the framework of the European Human Brain Project. The source code of the present tool, written in Python3, can be accessed at: https://github.com/lens-biophotonics/st_fibre_analysis_hbp. 3D representation of the vectors in Supplementary Fig. 7 was obtained using Mayavi (https://docs.enthought.com/mayavi/mayavi/misc.html)^35^.

In order to remove background and spurious dark regions, retaining only brain structures, and improve the reliability of the obtained vector fields, a threshold of 85% non-zero voxels was imposed beforehand on each 5-μm macro-voxel to be characterized.

In detail, local gradient-square tensors were first computed as the outer product of the image gradient $\nabla I$ with itself:

$$S_{v} \left( {x,y,z} \right) = \nabla I\nabla I^{T} = \left( {\begin{array}{*{20}c} {I_{x}^{2} } \\ {I_{x} I_{y} } \\ { I_{x} I_{z} } \\ \end{array} } \right.\left. {\begin{array}{*{20}c} {I_{x} I_{y} } & {I_{x} I_{z} } \\ {I_{y}^{2} } & {I_{y} I_{z} } \\ {I_{y} I_{z} } & {I_{z}^{2} } \\ \end{array} } \right),$$

where $I_{x}$ , $I_{y}$ and $I_{z}$ respectively denote the local first-order spatial derivatives along the x, y, and z axes. Tensor elements estimated voxel-wise were then averaged over 5-µm local neighborhoods after isotropic smoothing by means of Gaussian kernels $g_{{\sigma_{s} }}$ with standard deviation $\sigma_{s} = 3$ pixel.

3D tissue orientation maps were finally derived from the local directions of minimal gray level change, i.e. the eigenvector of the averaged structure tensor $\overline{S}_{{\sigma_{s} }}$ associated with the lowest eigenvalue.

### ODF calculation

Fiber orientation distribution functions (ODFs) were used to characterize a given distribution of 3D orientations, i.e., nerve fibers. To calculate the ODF of a given number of K orientation vectors, the individual spherical harmonic (SH) coefficients ${\text{c}}_{{{\text{lm}}}}$ of the ODF were estimated as ${\text{c}}_{{{\text{lm}}}} = \frac{{{\text{N}}_{{\text{l}}}^{{\text{m}}} }}{{\text{K}}}\mathop \sum \limits_{k = 1}^{K} P_{l}^{m} \cos \left( {\theta_{k} } \right)e^{{ - im\varphi_{k} }}$ with ${\text{N}}_{{\text{l}}}^{{\text{m}}}$ as the normalization coefficient, ${\text{P}}_{{\text{l}}}^{{\text{m}}}$ as Legendre polynomes, $\varphi$ as azimuthal, and ${\uptheta }$ as the polar angle. This calculation was applied to the orientations obtained in a super-voxel consisting of ${\text{n}}_{{\text{x}}} \times {\text{n}}_{{\text{y}}} \times {\text{n}}_{{\text{z}}}$ voxels. The visualization of the ODFs was done with the open-source tool *mrview* from MRtrix3 (https://mrtrix.readthedocs.io/en/latest/) ^26^. To avoid boundary artefacts, only super-voxels containing at least 1/3 of the total evaluated orientations were shown. The size of the ODFs was scaled by a factor of 2 to improve their visibility.

## Supplementary Information

Supplementary Information 1.

Supplementary Video 1.

Supplementary Video 2.

Supplementary Video 3.

Supplementary Video 4.

## Data Availability

All data supporting the findings of this study are included in figures and videos as representative images or data points in the plots. Additional images other than the representative images are available from the corresponding author upon reasonable request.

## Code availability

MRtrix3 is an open source tool; ZetaStitcher and STA codes are open source and available on GitHub at URLs provided in the Methods. The other custom code used in this study are available from the corresponding author upon reasonable request.
