# Supplementary material for: Autofluorescence enhancement for label-free imaging of myelinated fibers in mammalian brains
Source: Sci Rep. 2021 Apr 13;11:8038. doi: 10.1038/s41598-021-86092-7 (PMC8044204; doi:10.1038/s41598-021-86092-7)
Supplement: Supplementary file 1 — Supplementary Information 1. [file 41598_2021_86092_MOESM1_ESM.pdf]

Autofluorescence enhancement for label-free imaging of myelinated fibers in mammalian brains

Irene Costantini[1,2,3*], Enrico Baria[1,4], Michele Sorelli[1,4], Felix Matuschke[5], Francesco Giardini[1], Miriam Menzel[5], Giacomo Mazzamuto[3,1], Ludovico Silvestri[1,4], Riccardo Cicchi[3,1], Katrin Amunts[5,6], Markus Axer[5], Francesco Saverio Pavone[1,3,4]

[1]European Laboratory for Non-linear Spectroscopy, University of Florence, Italy,

[2]Department of Biology, University of Florence, Italy,

[3]National Institute of Optics, National Research Council, Italy,

[4]Department of Physics, University of Florence, Italy,

[5]Institute of Neuroscience and Medicine (INM-1), Research Centre Jülich, Jülich, Germany,

[6]C. and O. Vogt Institute for Brain Research, University Hospital Düsseldorf, Heinrich-Heine University Düsseldorf, Germany

*corresponding author costantini@lens.unifi.it

Supplementary information

1. Raman measurements and analysis

As reported in the main text, we used Raman spectroscopy for probing the glycerol content within myelinated fibers and the surrounding tissue before and after deglycerolization. In order to do so, we first recorded the Raman spectrum of the medium solution used for preparing and mounting glycerolized samples (20% glycerol, 2% DMSO, 4% formaldehyde). Supplementary figure S1a and S1b show the comparison between our measurement and the known spectra of glycerol and DMSO, respectively (spectra obtained from https://sdbs.db.aist.go.jp/sdbs/cgi-bin/cre_index.cgi). The main glycerol peaks can be easily identified in the recorded spectrum, while no major contribution can be attributed to the presence of DMSO. Then, we selected three major glycerol Raman bands (550, 850, and 1465 cm^{-1}) to be used for spectral projection: in fact, the “glycerol score” was calculated as the scalar product between each recorded spectrum and these

three bands of the spectrum. Hence, a score = 1 means a perfect match with the spectral signatures of glycerol, while a score < 1 implies lower abundance (or no presence at all) of that compound.

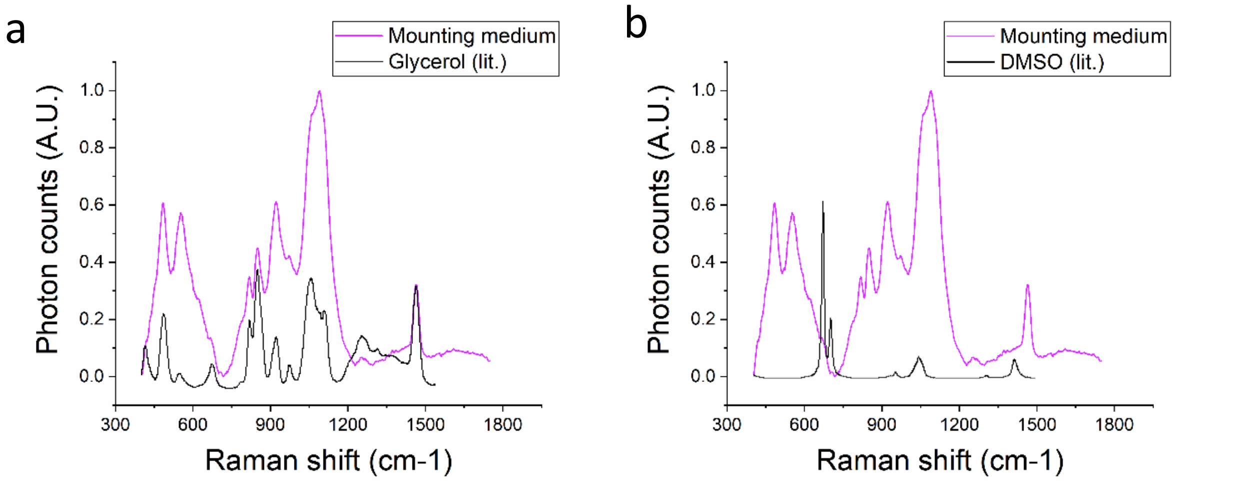

Supplementary Figure S1: Glycerol and DMSO Raman spectra. (a) Overlaying Raman spectra of mounting medium (magenta) and literature glycerol spectra (black). (b) Overlaying Raman spectra of mounting medium (magenta) and literature DMSO spectra (black). Graphs were prepared using OriginPro 9.0 (www.originlab.com).

2. Compatibility with one photon imaging

The MAGIC protocol was developed to help the study of myelinated fibers with fluorescence microscopy techniques. In order to demonstrate the versatility of the protocol, we performed acquisitions with a conventional confocal microscope at different wavelengths (Supplementary figure S2) and on different species (Supplementary figure S3).

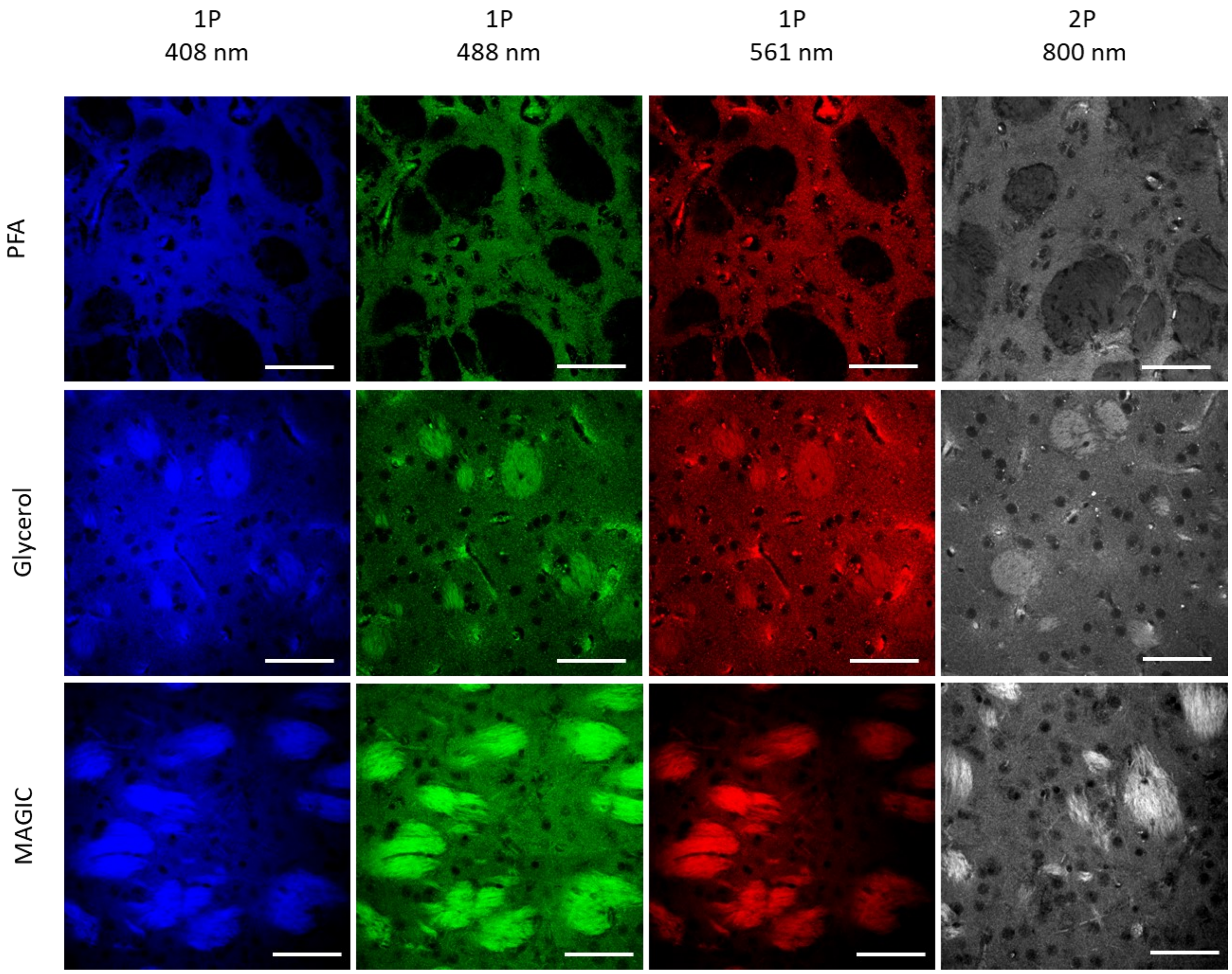

Supplementary Figure S2. One and two-photon excitation. Representative images of mouse brain sections during the three subsequent steps of the MAGIC protocol: fixation (PFA), glycerolization (Gly), and washing (MAGIC) acquired at different excitation wavelengths. One-photon (1P) excitation: 408, 488, 561nm; Two-photon (2P) excitation: 800nm. Scale bar = 50 µm. Images were prepared using Fiji (www.fiji.sc/Fiji).

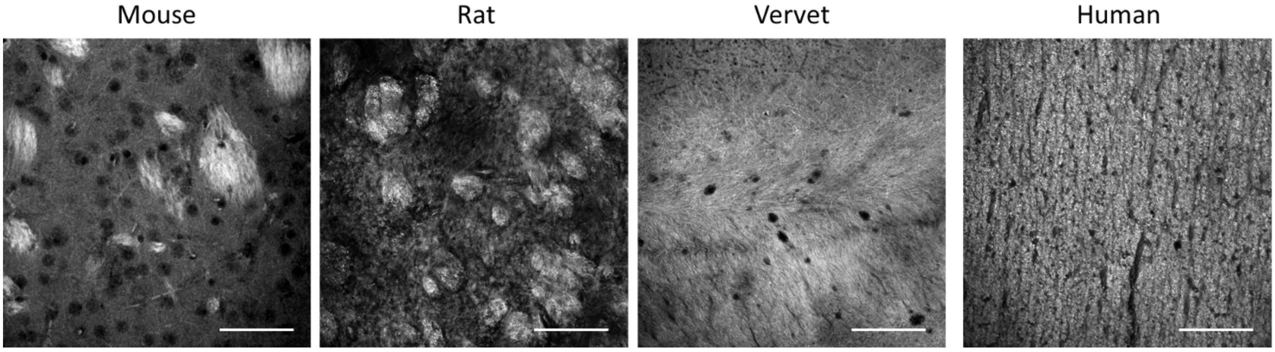

Supplementary Figure S3. One photon imaging of various species. Representative images of mouse, rat, vervet monkey, and human brain sections treated with MAGIC and acquired with the confocal microscope. Scale bar = 50 µm. Images were prepared using Fiji (www.fiji.sc/Fiji).

3. MAGIC protocol can provide details of myelinated axon substructures

To establish the possibility of studying the different organization of the myelin sheaths surrounding axons, we used a high magnification objective (Apo Plan Nikon 100× immersion oil objective) to detect the autofluorescence signal coming from them, as shown in Supplementary figure S4.

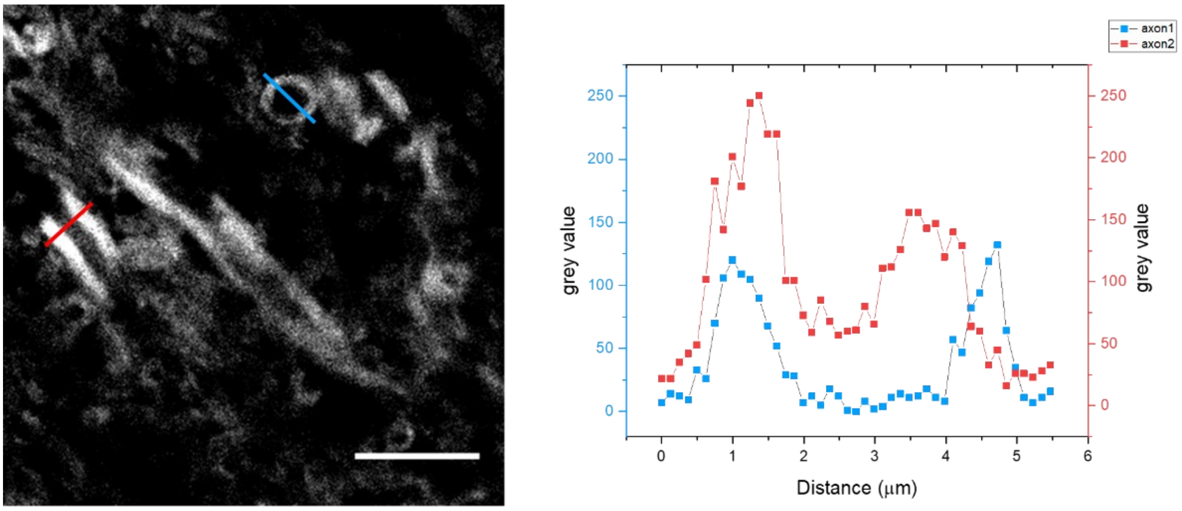

Supplementary Figure S4. Myelinated axon substructures. Image obtained with a 100x objective (λ_{exc}=488nm), Scale bar = 10 µm. The graph shows the intensity profile of the lines corresponding to the two axons (blue and red) of the image. Image and graph were prepared using Fiji (www.fiji.sc/Fiji).

4. MAGIC and Immunostaining

Representative images of human brain sections treated with MAGIC and stained with an anti-GFAP antibody are shown in Supplementary figure S5, and in Supplementary videos SV2 and SV3.

To perform the staining, the following protocol was applied. Brain sections previously treated with MAGIC protocol were permeabilized, incubating the samples for 45 min with PBS with 0.5% Triton X-100. After, samples were incubated with the primary antibody, an anti-GFAP antibody (abcam; ab7260) with a dilution of 1:200 in PBST 0.1% overnight at 4°C in the dark. The solution containing the primary antibody was removed and the sample was washed three times for 8 hours in PBST 0.1% solution. The day after, the sample was incubated with the secondary antibody, a donkey anti-rabbit IgG antibody conjugated with an alexa fluor 568 with a dilution of 1:200 (abcam, ab175470), in PBST 0.1% for 2 h at room temperature in the dark. The secondary antibody was then removed and the samples were washed three times with PBST 0.1% for 2h each in the dark at RT. Finally, the samples were washed twice with PBS for 10 min each at @RT, a coverglass was mounted on each section with transparent nailpolish, and TPFM imaging was performed.

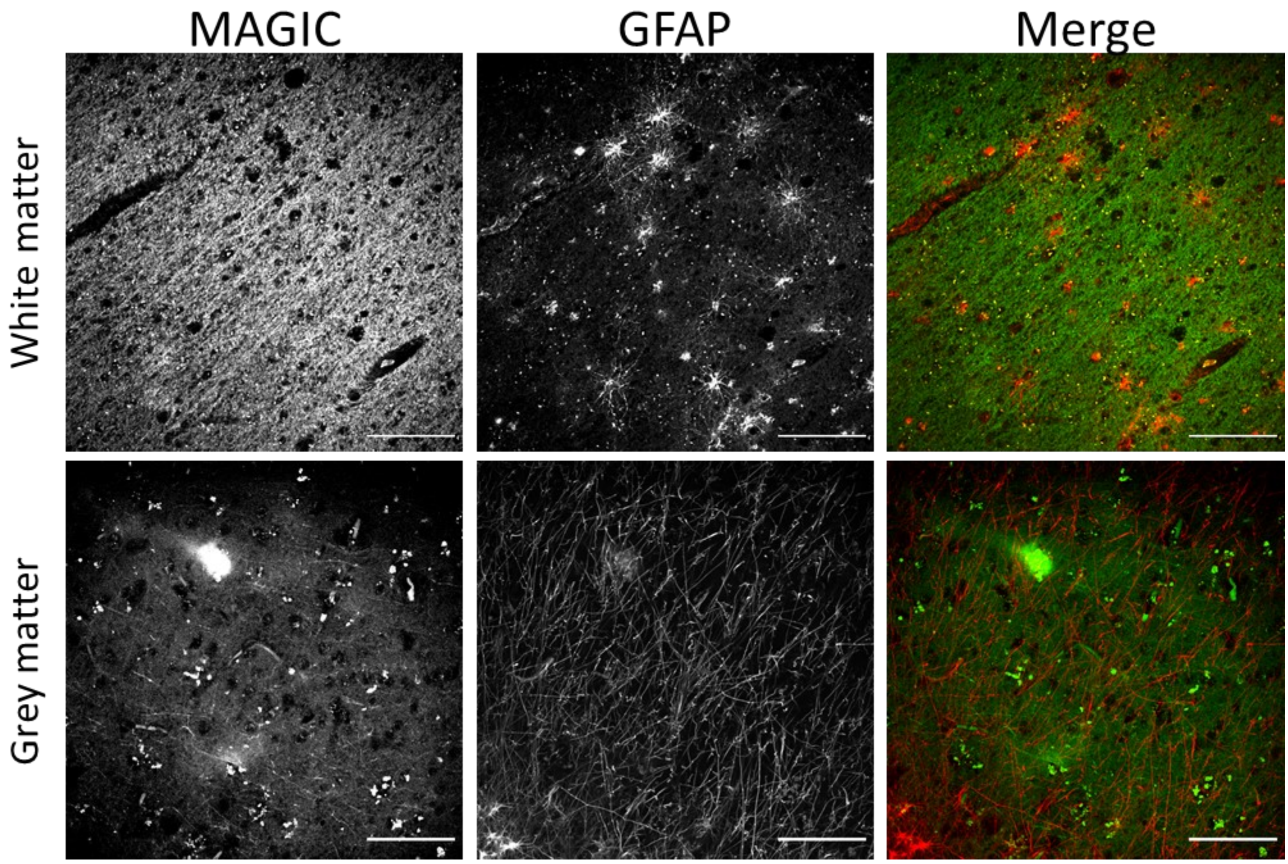

Supplementary Figure S5. Anti-GFAP Immunostaining. Images showing fibers (in green; MAGIC) and astrocytes (in red; anti-GFAP antibody) in both white (top row) and grey matter (bottom row) of a human brain section. Scale bar = 100 μm. Images were prepared using Fiji (www.fiji.sc/Fiji).

5. Control and Reeler fiber analysis pre-processing

In order to perform the analysis on different regions of interest of the control mouse (Control) and the Reeler mouse (Reeler), a manual segmentation of the areas was performed using Fiji (http://fiji.sc/Fiji). The Supplementary figure 6 shows the different masks superimposed on the maximum intensity projection (MIP) of the mosaic acquired with the TPFM.

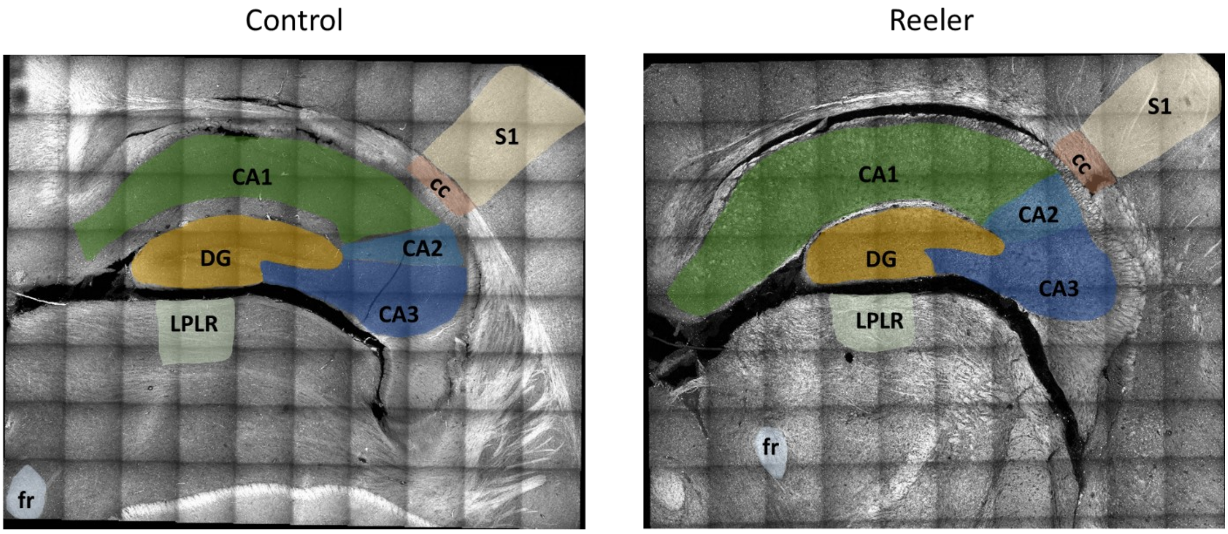

Supplementary Figure S6. Hippocampus ROIs. Masks of different areas superimposed to the MIP of the mosaic of the right hippocampal region of the control and the Reeler mouse sections. Acronym list = S1: Primary Somatosensory Cortex; cc: Corpus Callosum; CA1, CA2, CA3: field CA1, CA2, CA3 of hippocampus; DG: Dentate Gyrus ; LPLR: Lateral Posterior Thalamic Nucleus; fr: Fasciculus Retroflexus. Images and masks were prepared using Fiji (www.fiji.sc/Fiji).

6. Structure tensor analysis and orientation distribution functions evaluation

In order to compensate for the relative inclination of the brain sections with respect to the coronal image plane, the whole mesoscale stack mosaic of the hippocampus and its segmented ROIs were initially rotated along the frontal and longitudinal axes using Fiji. Next, image blurring was applied in the coronal plane so as to match the in-plane $FWHM_{XY}$ of the optical system PSF ($FWHM_{XY}$ = 0.692 µm) to the sagittal $FWHM_Z$ (2.612 µm). After these pre-processing operations, stack volumes were virtually decomposed into macro-voxels of (12, 12, 5) pixel size for obtaining a 5-µm tissue analysis resolution, and then downscaled within the image plane in order to set an isotropic spatial sampling period of 1 µm. As mentioned in the main text, a threshold of 85% non-zero voxels was applied to the separated 5-µm image sub-blocks with the aim to exclude the background and local regions associated with a scarce proportion of the brain tissue data. In practice, this macro-voxel selection step was instrumental in improving the accuracy of the resulting orientation estimates in boundary macro-voxels and in rejecting the contribution of spurious dark regions.

Afterwards, for each accepted macro-voxel, local (voxel-size) image gradient-structure tensors $S(x, y, z)$ were first computed as:

$$S(x,y,z) = \nabla I \nabla I^T * g_{\sigma_s} = \begin{pmatrix} I_x^2 * g_x & I_x I_y * g_y & I_x I_z * g_z \\ I_y I_x * g_x & I_y^2 * g_y & I_y I_z * g_z \\ I_z I_x * g_x & I_z I_y * g_y & I_z^2 * g_z \end{pmatrix},$$

where the first-order spatial derivates I_x , I_y and I_z were estimated using second-order accurate central differences, whereas g_x, g_y and g_z represent Gaussian smoothing filters with standard deviation $\sigma_s = 3$ pixel.

Local $S(x,y,z)$ elements were then averaged within each of the distinct 5-µm macro-voxels and the spectral decomposition reported below was performed:

$$\bar{S} = \begin{pmatrix} \bar{S}_{xx} & \bar{S}_{yx} & \bar{S}_{zx} \\ \bar{S}_{yx} & \bar{S}_{yy} & \bar{S}_{zy} \\ \bar{S}_{zx} & \bar{S}_{yz} & \bar{S}_{zz} \end{pmatrix} = \begin{pmatrix} | & | & | \\ v_1 & v_2 & v_3 \\ | & | & | \end{pmatrix} \begin{pmatrix} \lambda_1 & 0 & 0 \\ 0 & \lambda_2 & 0 \\ 0 & 0 & \lambda_3 \end{pmatrix} \begin{pmatrix} | & | & | \\ v_1 & v_2 & v_3 \\ | & | & | \end{pmatrix}^{-1}$$

(e-vectors) (e-values)

Finally, an eigenvalue analysis was conducted on the resulting decomposition for identifying the eigenvectors v_i associated with the lowest eigenvalue $\lambda_i < \lambda_j < \lambda_k$ ($i, j, k \in [1,2,3]$, axes), thus generating 3D tissue orientation maps corresponding to the directions of minimal gray level change at 5-um resolution. Orientation Distribution Functions ODFs evaluation was performed on the resulted vectors as described in the main text.

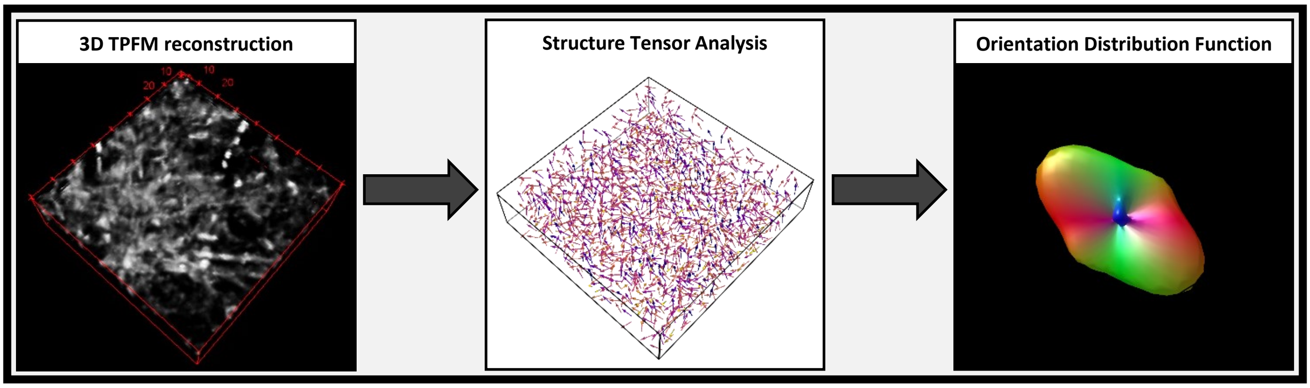

Supplementary Figure S7. Fibers' analysis pipeline. Block scheme representing the evaluation conducted on the TPFM images. The 3D rendering of the fluorescence image was prepared using Fiji (www.fiji.sc/Fiji), vectors maps using Mayavi (https://docs.enthought.com/mayavi/mayavi/misc.html), and ODF representations using MRtrix3 (https://mrtrix.readthedocs.io/en/latest/).

Supplementary figures legend:

Supplementary Figure S1: Glycerol and DMSO Raman spectra. (a) Overlaying Raman spectra of mounting medium (magenta) and literature glycerol spectra (black). (b) Overlaying Raman spectra of mounting medium (magenta) and literature DMSO spectra (black). Graphs were prepared using OriginPro 9.0 (www.originlab.com).

Supplementary Figure S2. One and two-photon excitation. Representative images of mouse brain sections during the three subsequent steps of the MAGIC protocol: fixation (PFA), glycerolization (Gly), and washing (MAGIC) acquired at different excitation wavelengths. One-photon (1P) excitation: 408, 488, 561nm; Two-photon (2P) excitation: 800nm. Scale bar = 50 µm. Images were prepared using Fiji (www.fiji.sc/Fiji).

Supplementary Figure S3. One photon imaging of various species. Representative images of mouse, rat, vervet monkey, and human brain sections treated with MAGIC and acquired with the confocal microscope. Scale bar = 50 µm. Images were prepared using Fiji (www.fiji.sc/Fiji).

Supplementary Figure S4. Myelinated axon substructures. Image obtained with a 100x objective (λ_{exc}=488nm), Scale bar = 10 µm. The graph shows the intensity profile of the lines corresponding to the two axons (blue and red) of the image. Image and graph were prepared using Fiji (www.fiji.sc/Fiji).

Supplementary Figure S5. Anti-GFAP Immunostaining. Images showing fibers (in green; MAGIC) and astrocytes (in red; anti-GFAP antibody) in both white (top row) and grey matter (bottom row) of a human brain section. Scale bar = 100 µm. Images were prepared using Fiji (www.fiji.sc/Fiji).

Supplementary Figure S6. Hippocampus ROIs. Masks of different areas superimposed to the MIP of the mosaic of the right hippocampal region of the control and the Reeler mouse sections. Acronym list = S1: Primary Somatosensory Cortex; cc: Corpus Callosum; CA1, CA2, CA3: field CA1, CA2, CA3 of hippocampus; DG: Dentate Gyrus ; LPLR: Lateral Posterior Thalamic Nucleus; fr: Fasciculus Retroflexus. Images and masks were prepared using Fiji (www.fiji.sc/Fiji).

Supplementary Figure S7. Fibers' analysis pipeline. Block scheme representing the evaluation conducted on the TPFM images. The 3D rendering of the fluorescence image was prepared using Fiji (www.fiji.sc/Fiji),

vectors maps using Mayavi (https://docs.enthought.com/mayavi/mayavi/misc.html), and ODF representations using MRtrix3 (https://mrtrix.readthedocs.io/en/latest/).

Supplementary videos legend:

SV1 Video1: Navigation in the 3D rendering of myelinated fibers after MAGIC. Imaging performed with TPFM, volume of 200 x 200 x 40 μm^3. The video was prepared using Amira 5.3 (ThermoFisher Scientific: www.thermofisher.com/it/en/home/industrial/electron-microscopy/electron-microscopy-instruments-workflow-solutions/3d-visualization-analysis-software/amira-life-sciences-biomedical.html).

SV2 Video2: 3D rendering navigation of a human brain white matter stack labeled with anti-GFAP after MAGIC. Imaging performed with TPFM: myelinated fibers in green and astrocytes in red. Volume of 450 x 450 x 60 μm^3. The video was prepared using Amira 5.3 (ThermoFisher Scientific: www.thermofisher.com/it/en/home/industrial/electron-microscopy/electron-microscopy-instruments-workflow-solutions/3d-visualization-analysis-software/amira-life-sciences-biomedical.html).

SV3 Video3: Rotation of a 150 x 150 x 60 μm^3 ROI of the video2 showing a single astrocyte. Imaging performed with TPFM: myelinated fibers in green and astrocytes in red. The video was prepared using Amira 5.3 (ThermoFisher Scientific: www.thermofisher.com/it/en/home/industrial/electron-microscopy/electron-microscopy-instruments-workflow-solutions/3d-visualization-analysis-software/amira-life-sciences-biomedical.html).

SV4 Video4: 3D rendering navigation of a representative stack of the human hippocampus after MAGIC. Imaging performed with TPFM: myelinated fibers in green and cell bodies in red due to lipofuscin autofluorescence. Volume of 450 x 450 x 60 μm^3. The video was prepared using Amira 5.3 (ThermoFisher Scientific: www.thermofisher.com/it/en/home/industrial/electron-microscopy/electron-microscopy-instruments-workflow-solutions/3d-visualization-analysis-software/amira-life-sciences-biomedical.html).
